# Supplementary material for: Effects of Simultaneous Exposure to a Western Diet and Wheel-Running Training on Brain Energy Metabolism in Female Rats
Source: Nutrients. 2021 Nov 26;13(12):4242. doi: 10.3390/nu13124242 (PMC8707360; doi:10.3390/nu13124242)
Supplement: Supplementary file 1 [file nutrients-13-04242-s001.zip › Figure S1 Heat map.pdf]

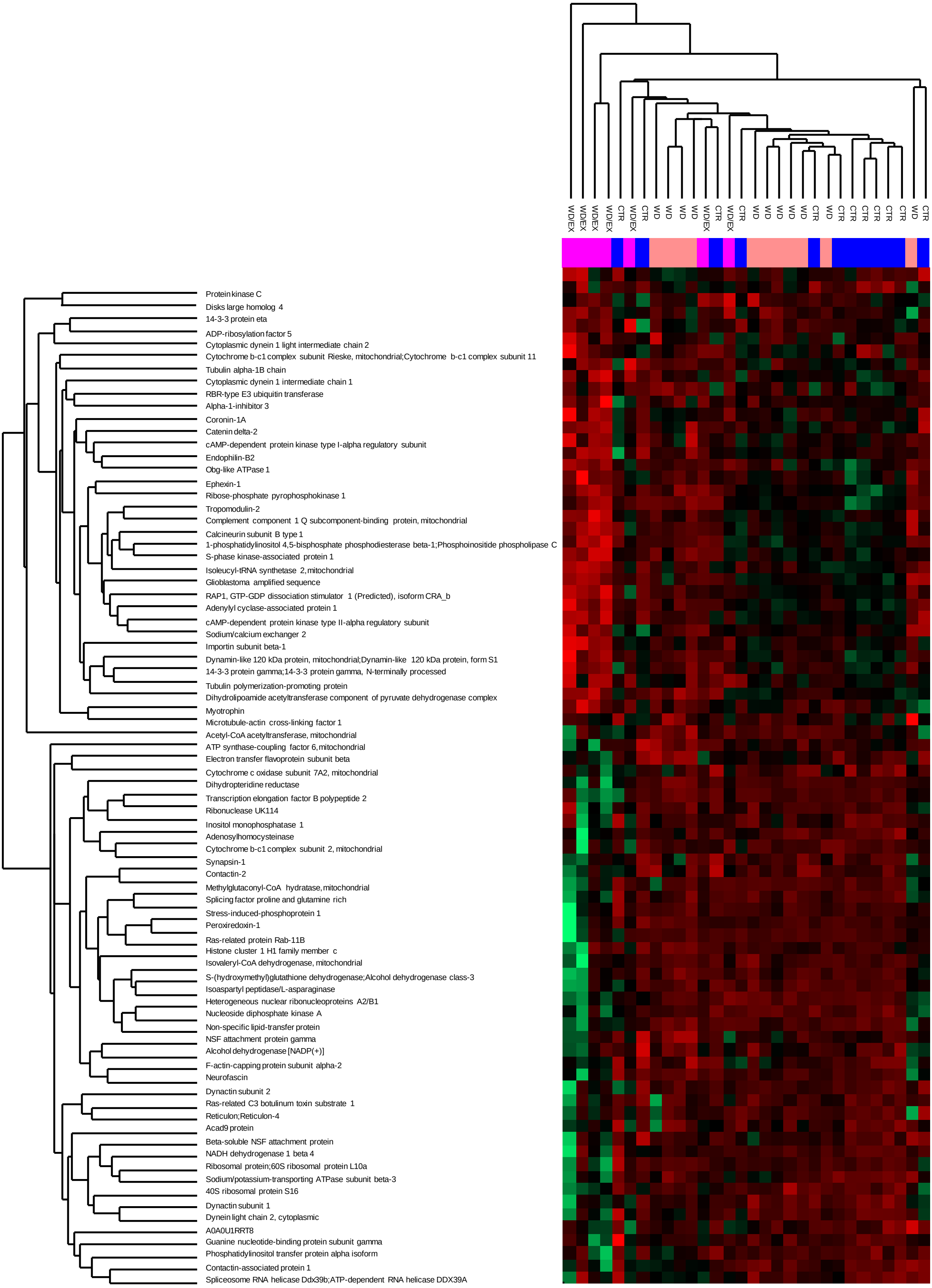

Figure S1. A heat map visualization of changes in proteomic profile of the temporal cortex among three experimental groups: control rats fed with standard diet (CTR, n = 12), rats fed with western diet (WD, n = 11), rats fed with western diet and exercised with wheel running (WD/EX, n = 7).
