# Supplementary material for: Effects of Simultaneous Exposure to a Western Diet and Wheel-Running Training on Brain Energy Metabolism in Female Rats
Source: Nutrients. 2021 Nov 26;13(12):4242. doi: 10.3390/nu13124242 (PMC8707360; doi:10.3390/nu13124242)
Supplement: Supplementary file 1 [file nutrients-13-04242-s001.zip › Table S1 A B Suplementary table western composition.pdf]

Table S1 A. Summary of macronutrient composition of the diets used in the present study.

Name	Standard diet (STD)	Western diet (WD) Set 1	Western diet (WD) Set 2
**Content**	Lobofeed B	Sausage<br>Crakers<br>Candy bar (Mars)	Tilsit cheese<br>Potato chips<br>Candy bar (Bounty)
**Total Kcal/g**	3.57	4.56	5.13
**FAT**			
**Kcal/g**	0.25	2.79	3.15
**% fat**	8%	31 %	35.3 %
**PROTEIN**			
**Kcal/g**	0.77	0.48	0.80
**% prot**	25%	12.8 %	20.4 %
**CARBOHYDRATE**			
**Kcal/g**	2.6	1.8	0.85
**% carb**	67%	45.1 %	21.4 %

Table S1 B. Western diet composition.

	Total kcal/100g	Protein g	Total fat g	Total Carb g	Dietary fiber g	Sodium g
**Polish dry sausage made of pork; Tarczyński**	611	26	55	3	0	3.3
**Tilsit cheese Hochland**	356	25.6	27.9	0.1	0	1.25
**potato chips; Lays Salt, Pepsci Co.**	526	32	52	5.9	1.4	4.4
**crakers (Lajkonik)**	476	8.8	21	62	1.7	2.3
**Candy bar Bounty (Mars Inc.)**	488	3.7	26.1	58.3	0	0.26
**Candy bar Mars (Mars Inc.)**	452	3.7	17	70.3	0	0.39
**10% fructose solution**	398	0	0	99.8	0	0
