# Supplementary material for: Effects of Simultaneous Exposure to a Western Diet and Wheel-Running Training on Brain Energy Metabolism in Female Rats
Source: Nutrients. 2021 Nov 26;13(12):4242. doi: 10.3390/nu13124242 (PMC8707360; doi:10.3390/nu13124242)
Supplement: Supplementary file 1 [file nutrients-13-04242-s001.zip › Table S2 List of antibodies used in western blot experiments.pdf]

Table S2. Table showing primary antibodies used in the experiment.

Antibody	Host animal	Dilution	Company(#catalog)
Acad9	rabbit, polyclonal	1:500	Abcam, #ab99952
Acat1	Produced recombinantly (animal-free) rabbit, monoclonal	1:1000	Abcam, #ab168342
ATP5j	Produced recombinantly (animal-free) rabbit, monoclonal	1:1000	Abcam, #ab181243
G6PD	rabbit, polyclonal	1:1000	Cell Signalling, #8866s
HK1	rabbit, monoclonal	1:1000	Cell Signalling, #2024s
MCT1	rabbit, polyclonal	1:1000	Abcam, #ab93048
MCT2	rabbit, polyclonal	1:1000	Abcam, #ab224627
MCT4	rabbit, polyclonal	1:750	Abcam, #ab74109
GLUT1	rabbit, polyclonal	1:1000	Abcam, #ab652
GLUT3	rabbit, polyclonal	1:1000	Abcam, #ab15311
GLUT8	Produced recombinantly (animal-free) rabbit, monoclonal	1:1000	Abcam, #ab169779
